# Lower back pain and associated factors among weavers working in Bahir Dar City, Northwest Ethiopia: A cross-sectional study

**DOI:** 10.3389/fpubh.2024.1400312

**Published:** 2024-05-21

**Authors:** Fiseha Sefiwu Zinabu, Kefale Getie, Mihret Dejen Takele, Samuel Teferi Chanie, Yohannes Abich, Yadelew Yimer Shibabaw, Alemu Kassaw Kibret

**Affiliations:** ^1^Department of Physiotherapy, School of Medicine, College of Medicine and Health Sciences, University of Gondar, Gondar, Ethiopia; ^2^Department of Medical Biochemistry, School of Medicine, College of Medicine and Health Sciences, University of Gondar, Gondar, Ethiopia

**Keywords:** lower back pain, musculoskeletal disorder, prevalence, associated factors, weavers

## Abstract

**Background:**

Work-related musculoskeletal disorders are widespread among workers of informal small-scale enterprises. Specifically, lower back pain is a prevalent occupational health problem across various industries, including weaving factories. Lower back pain significantly impairs the functioning, performance, and productivity of weavers. However, information on the prevalence and associated factors of low back pain among weavers of Bahir Dar City and nationwide is scarce. Therefore, this study aimed to assess the prevalence and associated factors of lower back pain among weavers working in Bahir Dar City.

**Method:**

A multicentered institutional-based cross-sectional study was conducted on 403 weavers in Bahir Dar City, Ethiopia, from April to May 2023 using a structured face-to-face interview questionnaire. Samples were proportionally allocated to each institution, and study participants were selected using a simple random sampling technique. Variables with a *P*-value < 0.25 in the bivariate logistic regression were adopted for the multivariate logistic regression analysis after verifying the model fitness. In the multivariate logistic regression analysis, the adjusted odds ratio (AOR) with a 95% confidence interval (CI) and a *P*-value < 0.05 were considered to identify the associated factors of lower back pain among weavers.

**Result:**

The results revealed that the overall annual prevalence of lower back pain was 63.5% (95% CI = 58.8–68.5). Longer working hours [AOR = 2.580 (CI = 1.517–4.384)], lack of back support [AOR = 1.938 (CI = 2.089–3.449)], repetitive movement of the back during weaving [AOR = 5.940 (CI = 2.709–13.02)], awkward posture [AOR = 2.915 (CI = 1.677–5.065), static working posture [AOR = 4.505 (CI = 2.298–8.831)], and job stress [AOR = 3.306 (CI = 1.896–5.765)] were significantly associated with lower back pain among weavers.

**Conclusions:**

Lower back pain among weavers was found to be highly prevalent. Working longer hours, lack of back support, repetitive movement of the back, awkward posture, static posture, and job stress were significantly associated with lower back among weavers. The study recommends prompt interventions on weavers to ensure that they use sitting support, weave for <8 h per day, change positions every 2 h, reduce job-related stress, and minimize task repetition, thereby enhancing their working conditions and minimizing the occurrence of lower back pain.

## Background

A musculoskeletal disorder (MSD) is a problem affecting the muscular system, which includes muscles, nerves, tendons, ligaments, joints, and cartilage, as well as the supporting structure of the neck, back, and other body parts (1, 2). Work-related MSDs are currently one of the most serious health problems that concern ergonomics at the workplace worldwide (3). They pose a significant threat to the health of employees in both developed and developing countries (4).

Lower back pain (LBP) is defined as pain localized between the 12th rib and the inferior gluteal folds, accompanied by or without leg pain (5). The symptoms can arise from various anatomical sources, including nerve roots, muscles, fascia, bones, joints, intervertebral discs, and organs in the abdominal cavity (6). LBP is the most common musculoskeletal issue worldwide (7, 8). Previous studies found that LBP caused an estimated 21.8 million disability-adjusted life years (9). According to a systematic review of the global prevalence of low back pain, approximately 60–80% of the people had LBP at some point in their lives (7). LBP creates a substantial personal, community, and financial burden worldwide (10–13). In addition, it causes activity limitation, absenteeism from work, and economic loss (14, 15).

The human back is particularly susceptible to injuries due to the complex mechanisms and various tissues and structures that comprise the spine (16). Several factors contribute to LBP among professionals, including poor posture, non-ergonomic way of working, and extended periods of working in the same position (15, 17). Prolonged periods of sitting without any change in posture can weaken abdominal muscles, causing spine curvature and respiratory and digestive disorders (18). Moreover, age, long hours of work (more than 8 h per day), frequent bending, years of work experience, awkward postures, repetitive movements, lack of back support, 7-day work weeks, job stress, and lack of job satisfaction are all factors associated with LBP (19–23). Efforts at workplaces to prevent LBP have gained increasing attention over the past years (24). In particular, exercise programs, ergonomic training programs, and physical and psychosocial interventions in workplaces have shown promise in reducing the incidence of LBP (25, 26).

Weaving is one of the world's oldest surviving crafts (27). It is an important cottage industry in developed and developing countries, including India, Pakistan, Bangladesh, Iran, Turkey, and China, where traditional weaving methods remain widely prevalent (28). Every year, over 1.5 million weavers, dyers, hand spinners, embroiderers, and other employees utilize over 0.3 million operating looms to produce 620 million metric tons of cloth (29). A handloom, which is constructed of wood and iron, is a manually operated machine or tool used for weaving fabric. Unlike electric motor-powered mechanisms, handlooms depend on the weavers' physical movement of the fabric using their hands and feet (30, 31).

People working in the weaving industry are extremely vulnerable to LBP due to a lack of occupational safety, insufficient health services, and poor working conditions. As a result, LBP has a detrimental effect on the functioning, performance, and productivity of weavers in Ethiopia are significantly contributing to the the national economy and development of the country. However, evidence on the prevalence of LBP and the factors affecting it among weavers in Bahir Dar City, Ethiopia, is still lacking. Therefore, this study aimed to assess the prevalence and associated factors of LBP among weavers by considering those working in Bahir Dar City, Ethiopia.

## Methods

### Study design and setting

A multicenter institutional-based cross-sectional study was conducted on weavers in Bahir Dar City, Northwest Ethiopia, from April to May 2023. According to the information obtained from the Bahir Dar City Bureau of Labor and Social Affairs, weavers in the city work either individually or under an institution. Our study participants were weavers working in an institution for more than 1 year. Bahir Dar is the capital city of the Amhara state, situated 565 km northwest of Addis Ababa, Ethiopia. The Ethiopian Central Statistics Agency estimated the city's population to be ~455,901 in 2022 (32). The city is located at 11° 35′ north latitude and 37° 23′ east longitude and 1,799 m or 5,902 feet above sea level (33).

### Eligibility criteria

#### Inclusion criteria

The study included weavers who were employed by an institution, above the age of 18 years, and had more than 1 year's working experience.

#### Exclusion criteria

The study excluded weavers with a history of LBP before weaving and those who had experienced trauma or undergone surgery within the 6 months before data collection.

### Sample size determination

The sample size (*n*) was calculated using a one population proportion formula, *n* = (*Z* α*/2*)^2^ × *p*(1– *p*))/*d*^2^ (22); under the assumption of a 48.9% prevalence (*p*) of LBP studies in Ethiopia, a 5% margin of error (*d*), and a 95% confidence interval (CI), we obtain n = ((1.96)^2^ × 0.489(1 – 0.489))/(0.05)^2^ = 384. Finally, by adding 10% of non-respondents, which is 38, the final sample size required for this study was 384 + 38 = 422.

### Sampling procedure and technique

**Figure 1:**
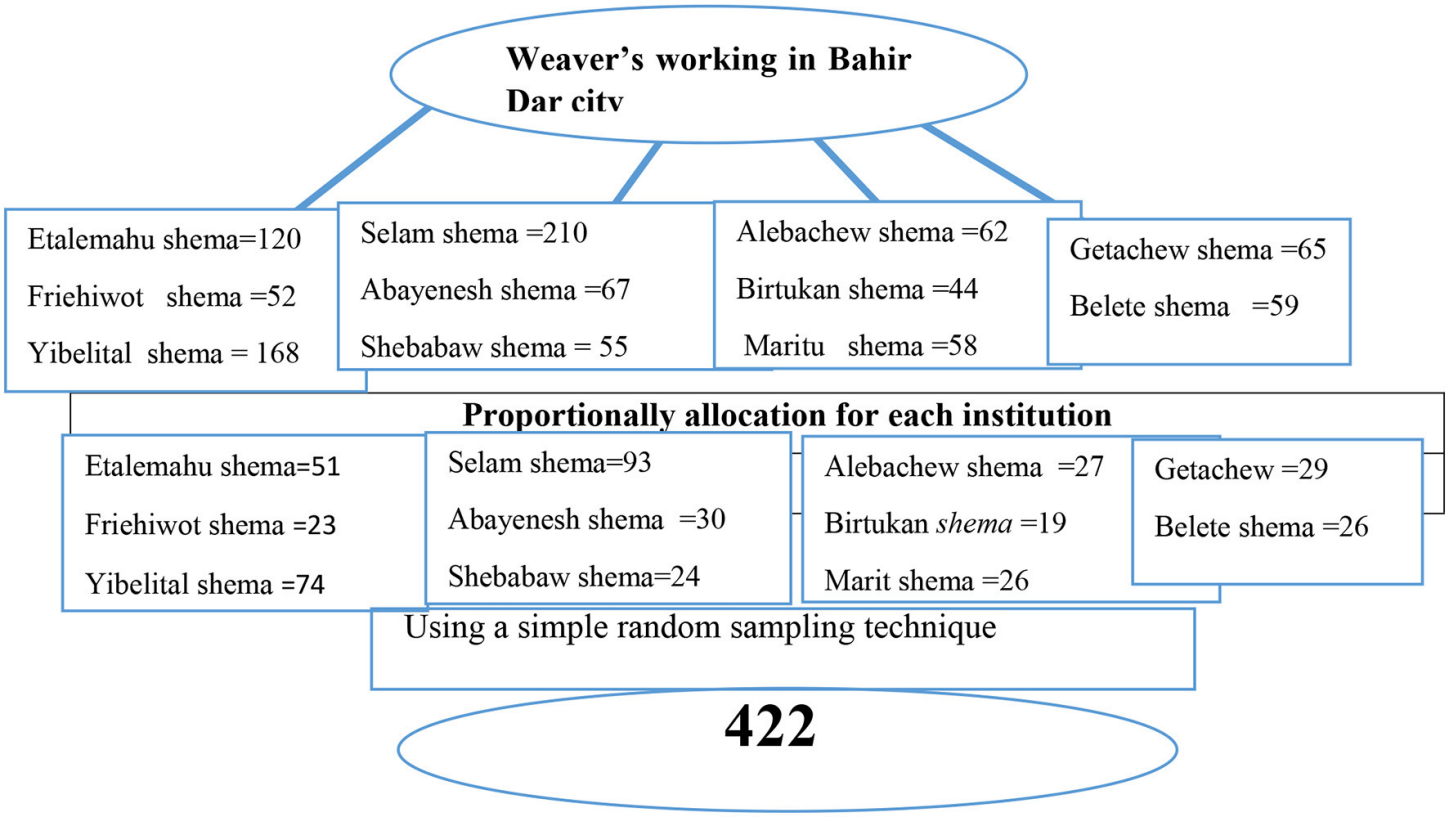
Schematic representation of the sampling procedure and technique of weavers working in Bahir Dar City, Northwest Ethiopia, 2023.

According to information from the Bahir Dar City Bureau of Labor and Social Affairs, the city has 11 recognized institutions that employ weavers. All institutions were included in the study and sampling frames were made based on the participant eligibility criteria. Finally, the sample size was proportionally allocated and participants were recruited using a simple random sampling technique (the lottery method; see Figure 1).

### Study variables

#### Dependent variable

Lower back pain (yes/no).

#### Independent variables

Personal and demographic characteristics—age, sex, body mass index (BMI), educational status, monthly family income, and the Oswestry Disability Index. Lifestyle and psychosocial factors—alcohol consumption, cigarette smoking, physical activity, job satisfaction, and job stress. Work-related factors—work experience, working hours, working days per week, repetitive movement of back, awkward posture, static posture, presence of back support, and breaks during work.

### Operational definitions

#### LBP

Individuals are considered to have LBP if they have experienced aches, pain, or discomfort localized below the costal margin and above the inferior gluteal folds during the last 12 months (34).

#### Awkward posture

A bent or twisted posture of the back of an individual during work (35).

#### Static posture

Posture of an individual sitting or standing in a restricted space for 2 or more hours without changing positions (36).

#### Repetitive movement

Individuals repeat the same motion of the lower back with little or no variation every few seconds for 2 or more hours (36).

#### BMI

The BMI is measured as weight in kilograms divided by the square of height in meters (kg/m^2^). Individuals are considered underweight if BMI < 18.50 kg/m^2^, normal weight if BMI = 18.50–24.99 kg/m^2^, overweight if BMI = 25–29.9 kg/m^2^, and obese if BMI ≥ 30 kg/m^2^ (37).

#### Smoker

Individuals who reported smoking daily (at least one cigarette per day) or occasionally (< 1 cigarette per day) are considered smokers (38).

#### Alcohol consumption

Individuals who drink beer, local beer, or Areke, Tella, or Tej every day or every other day are considered alcoholic (39).

#### Stress

Workers with a mean score of 12.5 and below were considered to have job stress based on five job stress-related questions scored on a four-point Likert scale (40).

#### Job satisfaction

Based on a five-point Likert scale, workers with a mean score of 15 and above were considered to be satisfied and those with a mean score below 15 were considered to be dissatisfied with their job (41).

#### Physical activity

Workers who engaged in at least 30 min of moderate to extensive physical activity each day for at least 3 days per week were considered to be physically active (42).

#### The Oswestry Disability Index

Researchers and disability evaluators typically measure a patient's permanent functional disability using the Oswestry Disability Index, which consists of 10 sections totaling 50 points and is scored on a Likert scale. The levels of disability of the individuals are defined as follows: a total percentage score ranging from zero to 20% indicates minimal disability, 20–30% indicates moderate disability, 40–60% is severe disability, 60–80% indicates crippled or handicapped, and 80–100% indicates bedbound (43).

### Data collection tool and procedures

To gather relevant data, a structured interview questionnaire adopted from the Nordic musculoskeletal questionnaire (44) and Oswestry Disability Index (43) was used. The questionnaire comprises three sections—Section I: sociodemographic characteristics, Section II: lifestyle and psychosocial characteristics, and Section III: work-related factors. Data collection was done by four physiotherapists and two supervisors, who were provided a 2-day training before data collection about the data collection tool, how to approach study participants, and the study's objective. A permission and support letter to conduct the study was prepared. Initially, a pretest was conducted for 5% (20 weavers) of the sample size in Dessie town, and necessary changes were made to the data collection tool for the actual study. The respondents were apprised about the importance of their responses for the study and they were requested to answer the questions with honesty.

### Data quality control

To ensure consistency, the questionnaire was first prepared in English, then translated into the local language Amharic, and, subsequently, backtranslated into English by bilingual experts. For the four data collectors and two supervisors to attain a common understanding and to ensure consistency and data quality, before the actual data collection, they were trained for 2 days about the data collection tool, how to approach participants, and conduct interviews. The questionnaire was pretested among Dessie town weavers. The supervisors evaluated the completed questionnaires on each day of data collection. After double-checking for consistency and completeness, the supervisors forwarded the completed surveys to the principal investigator. The questionnaires that were incomplete or had missing information were returned to data collectors for rectification. Supervisors rechecked 5% of the samples to ensure the data collectors completed their tasks correctly.

### Data analysis

The collected data were coded, cleaned, and entered into EpiData software version 4.6 and then exported to Statistical Package for Social Science (SPSS) version 25 packages for analysis. The results were analyzed using descriptive statistics, frequency, percentages, means, standard deviations, and logistic regression. Multicollinearity of the independent variables was checked by a variance inflation factor (VIF) cutoff point of < 10. Model fitness was analyzed by the Hosmer–Lemeshow test; a statistic >0.005 was considered a good fit. Variables with a *P*-value of < 0.25 in the bivariate logistic regression analysis were accepted as potential candidates in the final multivariate logistic regression analysis. The adjusted odds ratio (AOR) and 95% CI were employed to estimate and evaluate the predictors of LBP included in the multivariate logistic regression. In the final model, a *P*-value of < 0.05 and a 95% CI were considered statistically significant.

### Ethical consideration

Ethical approval was obtained from the Ethical Review Board of the School of Medicine (Ref No. 562), College of Medicine and Health Sciences, University of Gondar, by the Helenski declaration. A support letter was obtained from the Department of Physiotherapy, and permission to conduct the study was obtained from Amhara Regional Labor and Social Affairs. The purpose of the study was explained to the participants, and written and oral consent was obtained from each participant. Confidentiality was maintained and assured to the participants at all levels of the study. Participants' involvement in the study was voluntary. Participants who were unwilling to participate in the study and those who wished to quit their participation at any stage were permitted without any restrictions.

## Result

### Sociodemographic characteristics of study participants

Out of 422 weavers selected for the study, 403 weavers participated, yielding a response rate of 95.5%. Table 1 shows the sociodemographic characteristics of the participants. Of the study participants, 216 (53.6%) were men and the majority, 348 (86.4%), were aged < 35 years, with an average age and standard deviation of the study participants being 26.2 (±6.7). Approximately half of the study participants, 206 (51.1%), had an educational level above grade nine, and the majority, 269 (66.7%), had a family income of < ETB5,000.

**Table 1:** Sociodemographic characteristics of weavers working in Bahir Dar City, Northwest Ethiopia, in 2023 (*n* = 403).

**Variable**	**Frequency (*n*)**	**Percent (%)**
**Sex**		
Men	187	46.6
Women	216	53.6
**Age**		
18–35	348	86.4
≥35	55	13.6
**BMI (kg/m** ^2^ **)**		
Normal	329	81.6
Underweight	54	13.4
Overweight	20	5
**Educational status**		
Unable to write and read	48	11.9
Able to write and read	34	8.4
Grade 1–8	115	28.5
>9 grades	206	51.1
**Family monthly income (ETB)**		
≤ 5,000	269	66.7
5,000–10,000	107	26.6
≥10,000	27	6.7

### Work-related characteristics of study participants

Among the study respondents, 251 (62.3%) had been working more than 8 h each day, and the majority, 307 (76.2%), of the participants have < 5 years of working experience. All of them had a break time at some time in their working day and 295 (73.2%) worked 5 or 6 days per week. Additionally, 384 (670.5%) participants had a repetitive movement of the back while working. In terms of the respondents' ergonomic status, 276 (68.5%) had bad posture while working (Table 2).

**Table 2:** Work-related characteristics of weavers working in Bahir Dar City, Northwest Ethiopia, in 2023 (*n* = 403).

**Variables**	**Frequency (*n*)**	**Percent (%)**
**Job experience (year)**		
≤ 5	307	76.2
>5	96	23.8
**Working hours (h)**		
≤ 8	152	37.7
>8	251	62.3
**Working days**		
3 or 4	108	26.8
5 or 6	295	73.2
**Static posture**		
No	296	73.4
Yes	107	26.6
**Repetitive movement**		
No	119	29.5
Yes	284	70.5
**Back support**		
No	129	32
Yes	274	68.0
**Awkward posture**		
No	127	31.5
Yes	276	68.5
**Break time**		
No	0	0
Yes	410	100

### Lifestyle and psychosocial characteristics of study participants

In terms of the participants' lifestyle, 403 (100%) participants did not smoke and 285 (70.7%) did not drink alcohol. Moreover, 378 (93.8%) participants were not regularly involved in physical exercise, and 229 (55.3%) expressed job satisfaction (Table 3).

**Table 3:** Lifestyle and psychosocial characteristics of weavers working in Bahir Dar City, Northwest Ethiopia, in 2023 (*n* = 403).

**Variables**	**Frequency (*n*)**	**Percent (%)**
**Alcohol**		
No	285	70.7
Yes	118	29.3
**Physical exercise**		
No	378	93.8
Yes	25	6.2
**Job satisfaction**		
No	179	44.4
Yes	224	55.6
**Job stress**		
No	106	26.3
Yes	297	73.7

### Prevalence of LBP in association with the participant characteristics

The prevalence of LBP among weavers was 63.5%. Based on the Oswestry Disability Index scores, out of the total number of participants, 32.5% had minimal disabilities, 19.5% had moderate disabilities, 10.4% had severe disabilities, and 0.7% were crippled. Among the participants who experienced LBP, 126 (67.4%) were men and 130 (60.2%) were women. Furthermore, 225 (64.7%) were < 35 years of age, while 31 (56.6%) were over 35 years old. Of those who worked more than 8 h per day, 122 (72.5%) had LBP, whereas 74 (46.7%) participants who worked fewer than 8 h a day experienced LBP. The majority of the study participants [211 (74.3%)] who had repeated back movement during weaving experienced LBP (see Table 4).

**Table 4:** Prevalence of LBP with respect to different characteristics of the participants working in Bahir Dar City, Northwest Ethiopia, in 2023.

**Variables**	**Annual prevalence of LBP**			
	**No**		**Yes**	
	**Frequency (** ***n*** **)**	**Percent (%)**	**Frequency (** ***n*** **)**	**Percent (%)**
**Sex**				
Men	61	32.6	126	67.4
Women	86	39.8	130	60.2
**Age group**				
< 35	123	35.3	225	64.7
≥35	24	43.6	31	56.6
**BMI (kg/m** ^2^ **)**				
Normal	121	36.8	208	63.2
Underweight	17	31.5	37	68.5
Overweight	9	45	11	55
**Educational status**				
Unable to write and read	18	37.5	30	62.5
Able to write and read	13	38.2	21	61.8
Grade 1–8	48	41.7	67	58.3
Above grade 9	68	33	138	67
**Monthly income (ETB)**				
≤ 5,000	92	34.2	177	65.8
5,000–10,000	46	43	61	57
≥10,000	9	33.3	18	66.7
**Working experience (years)**				
≤ 5	118	38.4	189	61.6
>5	29	30.2	67	68.8
**Working hours per day (h)**				
≤ 8	78	51.3	74	48.7
≥8	69	27.5	182	72.5
**Working days per week**				
3 and 4	48	44.4	60	55.6
5 and 6	99	33.6	196	66.4
**Static posture**				
No	122	41.2	174	58.8
Yes	25	23.4	82	76.6
**Repetitive movement**				
No	74	62.2	45	37.8
Yes	73	25.7	211	74.3
**Back support**				
No	38	29.5	91	70.5
Yes	109	39.8	165	60.2
**Awkward posture**				
No	75	59.1	52	40.9
Yes	72	26.1	204	73.9
**Alcohol**				
No	90	31.6	195	68.4
Yes	57	48.3	61	51.7
**Physical activity**				
No	137	36.2	241	63.8
Yes	10	40	15	60
**Job satisfaction**				
No	69	38.5	110	61.5
Yes	78	34.8	146	65.2
**Job stress**				
No	56	52.8	50	47.2
Yes	91	30.6	206	69.4

### Associated factors of LBP among weavers

Bivariate and multivariate logistic regression models were used to analyze determinant factors. The results are shown in Table 5. In the bivariate logistic regression analysis, age, sex, alcohol, working experience, number of working days per week, working hours per day, repetitive movement, awkward posture, static posture, back support, and job stress were significantly associated with LBP. Finally, the following six variables showed statistically significant association with LBP in the multivariate logistic regression: working hours per day, lack of back support, repetitive movement, static posture, awkward posture, and job stress.

**Table 5:** Bivariate and multivariate logistic regression analysis of LBP among weavers working in Bahir Dar City, Northwest Ethiopia, in 2023 (*n* = 403).

**Variable**	**LBP**		**Bivariate**	**Multivariable**	
	**No (** ***n*** **)**	**Yes (** ***n*** **)**	**COR (95% CI)**	**AOR (95% CI)**	***P*** **-value**
**Age**					
< 35	123	225	1	1	
≥35	24	31	0.706 (0.39–1.25)^*^	0.582 (0.27–1.257)	0.168
**Sex**					
Men	61	126	1	1	
Women	86	130	0.732 (0.86–1.1)^*^	0.721 (0.408–1.274)	0.261
**Working experience**					
≤ 5	118	189	1	1	
>5	29	67	1.442 (0.88–2.36)^*^	1.380 (0.732–2.633)	0.329
**Working days**					
3 and 4	48	60	1	1	
5 and 6	99	196	1.568 (1.01–2.48)^*^	0.406 (0.186–0.885)	0.23
**Working hours**					
≤ 8	78	74	1	1	
>8	69	182	2.78 (1.82–4.22)^*^	2.580 (1.517–4.384)^**^	0.00
**Repetitive movement**					
No	74	45	1	1	0.00
Yes	73	211	4.75 (3.01–7.50)^*^	5.940 (2.709–13.02)^**^	
**Static posture**					
No	122	174	1	1	0.00
Yes	25	82	2.30 (2.38–3.80)^*^	4.505 (2.298–8.831)^**^	
**Back support**					
No	38	91	1.58 (1.01–2.48)	1.938 (2.089–3.449)^**^	0.024
Yes	209	165	1	1	
**Awkward posture**					
No	75	52	1	1	
Yes	72	204	4.07 (2.62–6.37)^*^	2.915 (1.677–5.065)^**^	0.00
**Alcohol**					
No	90	195	1	1	
Yes	57	61	0.494 (0.31–0.76)^*^	0.464 (0.296–0.801)	0.06
**Job stress**					
No	56	50	1	1	
Yes	91	206	2.535 (1.61–3.99)^*^	3.306 (1.896–5.765)^**^	0.00

^*^Significant at bivariate.

^**^Statistically significant at multivariate.

CI, confidence interval; COR, crude odds ratio; AOR, adjusted odds ratio; 1, reference.

The odds of acquiring LBP were 2.58 times greater among weavers who worked more than 8 h per day than among those who worked fewer than 8 h [AOR = 2.580, 95% CI (1.517–4.384)]. Weavers who did not utilize back support during work had 1.93 times higher odds of developing LBP than those who used back support [AOR = 1.938, 95% CI (2.089–3.449)]. Similarly, weavers who engaged in repeated movement of the back for more than 2 h during weaving were 5.94 times more likely to suffer LBP than those who did not have repeated movement [AOR = 5.940, 95% CI (2.709–13.02)]. In addition, weavers who had an awkward posture while weaving had 2.91 times higher odds of developing LBP [AOR = 2.915, 95% CI (1.677–5.065)]. Moreover, weavers with job stress had 3.30 times higher odds of acquiring LBP than those who did not have job stress [AOR = 3.306, 95% CI (1.896–5.765)]. The odds of experiencing LBP were 4.50 times higher among weavers who attained static posture [AOR = 4.505, 95% CI (2.298–8.831)] than those who changed their posture.

## Discussion

LBP, which is one of the most prevalent occupational health issues, is also linked to significant productivity loss and absenteeism from work. As a result, employers, workers, and the healthcare system bear financial costs related to LBP (45). Ethiopian weavers have focused on Ethiopia's status as the origin of handspun cotton, thereby substantially contributing to the country's economy (46). However, research in this field is scarce, and the health issues associated with this industry have not been paid attention. Therefore, this study aimed to assess the prevalence and associated factors of LBP among weavers working in Bahir Dar City.

In this study, the overall annual prevalence of LBP among weavers was found to be 63.5% (95% CI = 58.8–68.5), which is lower than the annual prevalence of LBP among weavers in Arunachal Pradesh and Varanasi in India, Northern Thailand, and Belgium (i.e., 79.2, 82.91, 71, and 81%, respectively) (21, 47–49). The variation may be because the mean age of the participants in the current study was less than those of the participants in these four studies [38.79 (±12.844), 54.65 (±10.41), 57.21 (±3.15), and 39 (±9) years, respectively]. Moreover, evidence supports that advancing age results in increased occurrences of work-related MSDs, including LBP (50). In addition, all participants of the study conducted in Arunachal Pradesh were women. This result could be because, compared with men, women are more likely to experience chronic pain problems and show greater sensitivity to unpleasant stimuli (51). Moreover, studies conducted in Varanasi, Northern Thailand, and Belgium showed a higher prevalence than that observed in the present study, which can be attributed to the fact that most participants had more than 5 years of working experience in these studies, contrary to the present study. This finding may be because longer years of working experience lead to a greater occurrence of pain in the body and increased pain intensity (4).

In addition, the results of the present study agree with the studies conducted in West Bengal, India (68%); Uttarakhand, India (67.19%); and the Gamo zone of Ethiopia (64.2%) (23, 52, 53). This similarity can be attributed to the comparable participant characteristics of the studies, such as age, sex, sample size, BMI, number of working hours per day, and assessment tools used.

The present study's findings revealed a higher prevalence than the studies conducted in Central and Southern Ethiopia (48.9%) and the Gulele Sub-city of Addis Ababa (50%) (19, 22). This disparity may be due to differences in the sex, age, working hours, and working area of the participants. In the studies conducted in Central and Southern Ethiopia, the participants were aged < 17 years, more than two-thirds of the participants were men, and more than two-thirds of the participants worked < 7 h per day. By contrast, in the Guile Sub-city study, most participants were men and the population included both individual households and cooperatives, which is different from the present study where the participants were working in institutions.

Weavers who work long hours per day were significantly associated with LBP. The results of the present study aligned with those of the studies conducted in Samarinda, Indonesia; Letmafo Induk Village, Insana Tengah District, Indonesia; and Belgium; these studies showed that longer daily working hours significantly raise the occurrence of LBP among weavers (21, 48, 54, 55). This outcome may be attributed to the wearing out of the workers' muscles and bones dye to long working hours, which results in reduced physical endurance, consequently leading to LBP (20). Compared to workers who used back support while working, those who did not use back support had an increased risk of LBP. In addition, the research on women textile workers conducted in Ethiopia and Belgium supports this finding (22, 56). The reason for the increased risk of LBP due to lack of back support could be the detrimental effects of posture, including elevated tension on the back muscles and intradiscal pressure (57).

Furthermore, weavers with repetitive movements for two or more hours had 5.94 times more likely higher odd of developing LBP. This study is supported by the study conducted in Belgium and India (21, 53). This finding could be due to the fact that repetitive actions at work exert a strain on the musculoskeletal system, which increases the risk of exhaustion and leads to insufficient time for tissue healing, thereby causing pain and discomfort (58, 59). Furthermore, participants who spent longer than 2 h weaving in a static posture had 4.50 times increased risk of developing LBP, which is supported by two studies conducted in India (52, 53). This finding can be attributed to the muscles' inability to relax and the obstruction of blood circulation when weavers work while maintaining a constant position (60).

Individuals who worked in an awkward posture were 2.91 times more likely to experience LBP than their counterparts. This result is supported by the research conducted in Belgium and India (21, 47). An awkward posture, characterized by the twisting or bending of the body, increases tension on joints, muscles, and nerves, causing tiredness and injury (61). Furthermore, weavers who experienced stress had 3.30 times higher odds of experiencing LBP, which is supported by the studies conducted in Ethiopia among young weavers and textile manufacturing workers (22, 62). This finding can be attributed to the fact that stress alters the human body and LBP is caused by prolonged and intense stress, which shortens micro-pauses in muscle action and tightens the muscles (63).

## Conclusion and recommendation

The present study demonstrated a high prevalence of LBP among weavers. Working longer hours, lack of back support, repetitive movement of the back, awkward posture, static posture, and job stress were significantly associated with LBP among weavers. We suggest that the health bureau highlight and support preventive measures, including educating legislators on LBP prevention and providing on-the-job training in the weaving field. Furthermore, we recommend prompt interventions for weavers, such as utilizing sitting support, limiting weaving work to < 8 h per day, switching positions every 2 h, reducing job-related stress, and minimizing repetitive tasks, to enhance working conditions and thus minimize the occurrence of LBP. We recommend conducting observational research, utilizing longitudinal study designs, and using objective measurements to investigate further factors associated with the prevalence of LBP among weavers.

## Limitations of the study

The study was based on a cross-sectional study design, which does not elucidate the temporal relationship between LBP and factors affecting its occurrence. In addition, the Nordic Musculoskeletal Disorder Questionnaire used in the study cannot identify the degree of LBP symptoms at risk. Furthermore, as the study subjects were limited to the weavers working in institutions, the results of the study cannot be applied to a general population.

## Data availability statement

The original contributions presented in the study are included in the article/supplementary material, further inquiries can be directed to the corresponding author upon reasonable request: fisehaseifu44@gmail.com.

## Ethics statement

The studies involving humans were approved by University of Gondar, College of Medicine and Health Sciences. The studies were conducted in accordance with the local legislation and institutional requirements. Written informed consent for participation in this study was provided by the participants' legal guardians/next of kin.

## Author contributions

FZ: Conceptualization, Data curation, Formal analysis, Investigation, Methodology, Resources, Supervision, Writing – original draft, Writing – review & editing. KG: Methodology, Conceptualization, Writing – review & editing. MT: Conceptualization, Writing – review & editing. SC: Data curation, Writing – original draft. YS: Conceptualization, Supervision, Writing – review & editing. AK: Data curation, Methodology, Conceptualization, Formal analysis, Writing – original draft, Writing – review & editing. YA: Data curation, Investigation, Writing – review & editing.

## References

[1] Skamagki G Carpenter C King A Wåhlin C. How do employees with chronic musculoskeletal disorders experience the management of their condition in the workplace? A metasynthesis. J. Occup. Rehabil. (2023) 2023:1–11. 10.1007/s10926-023-10099-2 36849842 PMC10684637

[2] Dehghan P Arjmand N. The National Institute for Occupational Safety and Health (NIOSH) recommended weight generates different spine loads in load-handling activity performed using stoop, semi-squat and full-squat techniques; a full-body musculoskeletal model study. Hum. Fact. 2022:187208221141652. 10.1177/00187208221141652 36433743

[3] Kodle NR Bhosle SP Pansare VB. Ergonomic risk assessment of tasks performed by workers in granite and marble units using ergonomics tool's REBA. Mater. Tod. (2023) 72:1903–16. 10.1016/j.matpr.2022.10.153

[4] Banerjee P Gangopadhyay S. A study on the prevalence of upper extremity repetitive strain injuries among the handloom weavers of West Bengal. J Hum Ergol. (2003) 32:17–22. 15176126

[5] Krismer M Van Tulder M. Low back pain (non-specific). Best Pract. Res. Clin. Rheumatol. (2007) 21:77–91. 10.1016/j.berh.2006.08.004 17350545

[6] Garland EL. Pain processing in the human nervous system: a selective review of nociceptive and biobehavioral pathways. Prim. Care. (2012) 39:561–71. 10.1016/j.pop.2012.06.013 22958566 PMC3438523

[7] Hoy D Bain C Williams G March L Brooks P Blyth F. A systematic review of the global prevalence of low back pain. Arthrit. Rheumat. (2012) 64:2028–37. 10.1002/art.34347 22231424

[8] Maher C Underwood M Buchbinder R. Non-specific low back pain. Lancet. (2017) 389:736–47. 10.1016/S0140-6736(16)30970-9 27745712

[9] Hoy D March L Brooks P Woolf A Blyth F Vos T. Measuring the global burden of low back pain. Best Pract. Res. Clin. Rheumatol. (2010) 24:155–65. 10.1016/j.berh.2009.11.002 20227638

[10] Manchikanti L. Epidemiology of low back pain. Pain Physician. (2000) 3:167–92. 10.36076/ppj.2000/3/167 16906196

[11] Dionne CE Dunn KM Croft PR. Does back pain prevalence really decrease with increasing age? A systematic review. Age Ageing. (2006) 35:229–34. 10.1093/ageing/afj055 16547119

[12] Rapoport J Jacobs P Bell NR Klarenbach S. Refining the measurement of the economic burden of chronic diseases in Canada. Age. (2004) 20:1–643. 15298484

[13] Deyo RA Cherkin D Conrad D Volinn E. Cost, controversy, crisis: low back pain and the health of the public. Annu Rev Public Health. (1991) 12:141–56. 10.1146/annurev.pu.12.050191.001041 1828670

[14] Lee H Hübscher M Moseley GL Kamper SJ Traeger AC Mansell G. How does pain lead to disability? A systematic review and meta-analysis of mediation studies in people with back and neck pain. Pain. (2015) 156:988–97. 10.1097/j.pain.0000000000000146 25760473

[15] Driscoll T Jacklyn G Orchard J Passmore E Vos T Freedman G. The global burden of occupationally related low back pain: estimates from the Global Burden of Disease 2010 study. Ann Rheum Dis. (2014) 73:975–81. 10.1136/annrheumdis-2013-204631 24665117

[16] Mallapiang F Muis M. The relationship of posture working with musculoskeletal disorders (MSDs) in the weaver West Sulawesi Indonesia. Gaceta Sanitaria. (2021) 35:S15–S8. 10.1016/j.gaceta.2020.12.005 33832616

[17] Oakman J Clune S Stuckey R. Work-Related Musculoskeletal Disorders in Australia. Canberra, ACT: Safe Work Australia (2019).

[18] Sun Y Nimbarte AD Motabar H. Physical risk factors associated with the work-related neck/cervical musculoskeletal disorders: a review. Indus Syst Eng Rev. (2017) 5:44–60. 10.37266/ISER.2017v5i1.pp44-60

[19] Terfe A Jemal T Waqkene T. Prevalence of low back pain and its associated factors among traditional cloth weavers in Gulele sub-city, Addis Ababa, Ethiopia. Front Publ Health. (2023) 11:1181591. 10.3389/fpubh.2023.1181591 37521989 PMC10374213

[20] Suryati Y Nggarang BN. Analysis of working postures on the low back pain incidence in traditional Songket Weaving Craftsmen in Ketang Manggarai Village, NTT. J Epidemiol Publ Health. (2020) 5:469–76. 10.26911/jepublichealth.2020.05.04.09

[21] Kaboré PA Schepens B. Work-related musculoskeletal disorders and risk factors among weavers: a cross-sectional study. South Afr J Physiother. (2023) 79:1904. 10.4102/sajp.v79i1.1904 PMC11157636 38855078

[22] Tefera Zele Y Ahmed AN Wondie Y Yilma TM Gebreegziabher HW Weldegebreal MK. Prevalence of low back pain and associated factors among young workers in traditional weaving of the informal sectors, Central and Southern Ethiopia. Vulner Child Youth Stud. (2020) 15:48–59. 10.1080/17450128.2019.1683926

[23] Haftu D Kerebih H Terfe A. Prevalence of work-related musculoskeletal disorders and its associated factors among traditional cloth weavers in Chencha district, Gamo zone, Ethiopia, an ergonomic study. PLoS ONE. (2023) 18:e0293542. 10.1371/journal.pone.0293542 37943828 PMC10635530

[24] Al-Otaibi ST. Prevention of occupational back pain. J Fam Commun Med. (2015) 22:73–7. 10.4103/2230-8229.155370 25983601 PMC4415130

[25] Hurwitz EL Randhawa K Yu H Côté P Haldeman S. The Global Spine Care Initiative: a summary of the global burden of low back and neck pain studies. Eur Spine J. (2018) 27:796–801. 10.1007/s00586-017-5432-9 29480409

[26] Bell JA Burnett A. Exercise for the primary, secondary and tertiary prevention of low back pain in the workplace: a systematic review. J Occup Rehabil. (2009) 19:8–24. 10.1007/s10926-009-9164-5 19219537

[27] Pandit S Kumar P Chakrabarti D. Ergonomic problems prevalent in handloom units of North East India. Age. (2013) 26:18–35.

[28] Hossain A. Prevalence of Musculoskeletal Disorder Among the Hand Loom Workers. Savar: Bangladesh Health Professions Institute, Faculty of Medicine, the University (2016).

[29] Tang KHD. A review of ergonomic intervention programs to reduce the prevalence of musculoskeletal disorders. Glob. Acad. J. Med. Sci. (2021) 3:183–90. 10.36348/gajms.2021.v03i05.007

[30] Khan M Momin A. Role of Handloom Board to Generate Employment in Rural Area: a Study of Enaitpur Thana in Sirajgonj. Dhaka: BRAC University (2013).

[31] Singh BP Kumar B Devi A. Traditional handloom weaving: a cultural heritage in jeopardy among Gaddi scheduled tribe of Bhaderwah (J&K), North West Himalayas. IJAR. (2021) 7:24–31.

[32] Amhara National Regional State. Population. (2009). Available online at: https://bahirdarcity.net/population.html (accessed February 17, 2019).

[33] DATEANDTIME.INFO. Geographical Coordinates of Bahir Dar, Ethiopia. (2019). Available online at: http://dateandtime.info/citycoordinates.php?id=342884 (accessed January 22, 2019).

[34] Workneh BS Mekonen EG. Prevalence and associated factors of low back pain among bank workers in Gondar City, Northwest Ethiopia. Orthoped Res Rev. (2021) 2021:25–33. 10.2147/ORR.S300823 33603503 PMC7881777

[35] Maxwell JT. Ergonomics Within the Workplace: an Occupation Based Injury Prevention Program for Computer Users. Eastern Kentucky University (2017).24346267

[36] Richard K. Prevalence of and Risk Factors for Work-related Musculoskeletal Injuries (WMSIs) Amongst Underground Mine Workers in Kitwe, Zambia. Cape Town: University of the Western Cape (2008).

[37] Tan K. Appropriate body-mass index for Asian populations and its implications for policy and intervention strategies. Lancet. (2004) 363:157–63. 10.1016/S0140-6736(03)15268-3 14726171

[38] Organization WH. Guidelines for Controlling and Monitoring the Tobacco Epidemic. Geneva: World Health Organization (1998).

[39] Yosef T Belachew A Tefera Y. Magnitude and contributing factors of low back pain among long distance truck drivers at Modjo dry port, Ethiopia: a cross-sectional study. J Environ Publ Health. (2019) 2019:6793090. 10.1155/2019/6793090 31662767 PMC6778925

[40] Aweto HA Tella BA Johnson OY. Prevalence of work-related musculoskeletal disorders among hairdressers. Int J Occup Med Environ Health. (2015) 28:545–55. 10.13075/ijomeh.1896.00291 26190730

[41] Nag A Vyas H Nag P. Gender differences, work stressors and musculoskeletal disorders in weaving industries. Ind Health. (2010) 48:339–48. 10.2486/indhealth.48.339 20562510

[42] World Health Organization. Global Recommendations on Physical Activity for Health. Geneva: World Health Organization (2010). 26180873

[43] Fairbank JC Pynsent PB. The Oswestry disability index. Spine. (2000) 25:2940–53. 10.1097/00007632-200011150-00017 11074683

[44] Kuorinka I Jonsson B Kilbom A Vinterberg H Biering-Sørensen F Andersson G. Standardised Nordic questionnaires for the analysis of musculoskeletal symptoms. Appl Ergon. (1987) 18:233–7. 10.1016/0003-6870(87)90010-X 15676628

[45] Harkness E MacFarlane GJ Nahit E Silman A McBeth J. Risk factors for new-onset low back pain amongst cohorts of newly employed workers. Rheumatology. (2003) 42:959–68. 10.1093/rheumatology/keg265 12730508

[46] Temesgen AG Tursucular O Eren R Ulcay Y. The art of hand weaving textiles and crafting on socio-cultural values in Ethiopian. Int J Adv Multidiscipl Res. (2018) 5:59–67.

[47] Mica N. Work-related musculoskeletal disorders among traditional weavers of districts of Arunachal Pradesh—a cross sectional study. Ind J Physiother Occup Ther. (2021) 15:16165. 10.37506/ijpot.v15i3.16165

[48] Siddiqui LA Banerjee A Chokhandre P Unisa S. Prevalence and predictors of musculoskeletal disorders (MSDs) among weavers of Varanasi, India: a cross-sectional study. Clin Epidemio Glob Health. (2021) 12:100918. 10.1016/j.cegh.2021.100918

[49] Chantaramanee N Taptagaporn S Piriyaprasarth P. The assessment of occupational ergonomic risks of handloom weaving in northern Thailand. Sci Technol Asia. (2015) 2015:29–37.

[50] Bernard BP Putz-Anderson V. Musculoskeletal Disorders and Workplace Factors; a Critical Review of Epidemiologic Evidence for Work-Related Musculoskeletal Disorders of the Neck, Upper Extremity, and Low Back (1997).

[51] Fillingim RB. Sex-related influences on pain: a review of mechanisms and clinical implications. Rehabil Psychol. (2003) 48:165. 10.1037/0090-5550.48.3.16510998712

[52] Naz H Kwatra S Ojha P. Prevalence of musculoskeletal disorders among handloom weavers of Uttarakhand: an ergonomic study. J Appl Nat Sci. (2015) 7:102–5. 10.31018/jans.v7i1.571

[53] Durlov S Chakrabarty S Chatterjee A Das T Dev S Gangopadhyay S. Prevalence of low back pain among handloom weavers in West Bengal, India. Int J Occup Environ Health. (2014) 25224808 10.1179/2049396714Y.0000000082 PMC4164884

[54] Ramdan IM Candra KP Fitri AR. Factors affecting musculoskeletal disorder prevalence among women weavers working with handlooms in Samarinda, Indonesia. Int J Occup Saf Ergon. (2018) 26:507–13. 10.1080/10803548.2018.1481564 29889000

[55] Ruliati LP Limbu R Sopbaba A. Ergonomic Risks Associated with Musculoskeletal Disorders in Ikat Weaving Workers in Letmafo Induk Village, Insana Tengah District, Timor Tengah Utara Regency (2021).

[56] Metgud D Khatri S Mokashi M Saha P. An ergonomic study of women workers in a woolen textile factory for identification of health-related problems. Ind J Occup Environ Med. (2008) 12:14–9. 10.4103/0019-5278.40810 20040992 PMC2796762

[57] Simpson T. A review of: “*Ergonomics, Work and Health*”, by STEPHEN PHEASANT, Macmillan Press, Basingstoke (1991), pp. x+ 358, £ 19 99, ISBN 0-333-48998-5. Ergonomics. (1994) 37:1128–9. 10.1080/00140139408963725

[58] Tiwari RR Pathak MC Zodpey SP. Low back pain among textile workers. Ind J Occup Environ Med. (2003) 7:27–9. 10.4103/0973-2284.15251

[59] Nag A Vyas H Nag P. Occupational health scenario of Indian informal sector. Ind Health. (2016) 54:377–85. 10.2486/indhealth.2015-0112 26903262 PMC4963551

[60] Luttmann A Jager M Griefahn B Caffier G Liebers F Organization WH. Preventing Musculoskeletal Disorders in the Workplace. WHO (2003).

[61] Etana G Ayele M Abdissa D Gerbi A. Prevalence of work related musculoskeletal disorders and associated factors among bank staff in Jimma city, Southwest Ethiopia, 2019: an institution-based cross-sectional study. J Pain Res. (2021) 2021:2071–82. 10.2147/JPR.S299680 34267551 PMC8275204

[62] Aderaw Z Engdaw D Tadesse T. Determinants of occupational injury: a case control study among textile factory workers in Amhara Regional State, Ethiopia. J Trop Med. (2011) 2011. 10.1155/2011/657275 22174723 PMC3235897

[63] Maduagwu SM Maijindadi RD Duniya KI Oyeyemi AA Saidu IA Aremu BJ. Prevalence and patterns of work-related musculoskeletal disorders among bankers in Maiduguri, Northeast Nigeria. Occup Med Health Aff . (2014) 2014:1–6. 10.4172/2329-6879.1000169

